# Histone deacetylases modulate resistance to the therapy in lung cancer

**DOI:** 10.3389/fgene.2022.960263

**Published:** 2022-10-03

**Authors:** Estefanía Contreras-Sanzón, Heriberto Prado-Garcia, Susana Romero-Garcia, David Nuñez-Corona, Blanca Ortiz-Quintero, Cesar Luna-Rivero, Victor Martínez-Cruz, Ángeles Carlos-Reyes

**Affiliations:** ^1^ Posgrado de Ciencias Genómicas, Universidad Autónoma de la Ciudad de México, Ciudad de México, México; ^2^ Laboratorio de Onco-Inmunobiologia, Departamento de Enfermedades Crónico-Degenerativas, Instituto Nacional de Enfermedades Respiratorias Ismael Cosio Villegas, Ciudad de México, México; ^3^ Facultad de Ciencias, Universidad Nacional Autónoma de México, Ciudad de México, México; ^4^ Departamento de Investigación en Bioquímica, Unidad de Investigación, Instituto Nacional de Enfermedades Respiratorias Ismael Cosio Villegas, Ciudad de México, México; ^5^ Servicio de Patología, Instituto Nacional de Enfermedades Respiratorias Ismael Cosio Villegas, Ciudad de México, México; ^6^ Laboratorio de Biología Molecular, Instituto Nacional de Pediatría, Ciudad de México, México

**Keywords:** lung cancer, histone deacetylases (HDACs), HDACs inhibitors, resistance to therapy, natural compounds

## Abstract

The acetylation status of histones located in both oncogenes and tumor suppressor genes modulate cancer hallmarks. In lung cancer, changes in the acetylation status are associated with increased cell proliferation, tumor growth, migration, invasion, and metastasis. Histone deacetylases (HDACs) are a group of enzymes that take part in the elimination of acetyl groups from histones. Thus, HDACs regulate the acetylation status of histones. Although several therapies are available to treat lung cancer, many of these fail because of the development of tumor resistance. One mechanism of tumor resistance is the aberrant expression of HDACs. Specific anti-cancer therapies modulate HDACs expression, resulting in chromatin remodeling and epigenetic modification of the expression of a variety of genes. Thus, HDACs are promising therapeutic targets to improve the response to anti-cancer treatments. Besides, natural compounds such as phytochemicals have potent antioxidant and chemopreventive activities. Some of these compounds modulate the deregulated activity of HDACs (e.g. curcumin, apigenin, EGCG, resveratrol, and quercetin). These phytochemicals have been shown to inhibit some of the cancer hallmarks through HDAC modulation. The present review discusses the epigenetic mechanisms by which HDACs contribute to carcinogenesis and resistance of lung cancer cells to anticancer therapies.

## 1 Introduction

Lung cancer is the leading cause of death from cancer worldwide. The estimated rate is about 2.3 million new cases per year and 1.8 million deaths within the same year (Sung et al., 2021). Non-small cell lung cancer (NSCLC) accounts for 85% of cases, while small cell lung cancer (SCLC) accounts for the rest. Patients with advanced-stage NSCLC have a poor survival time, which is lower than 5 years. This is because of relapse and the development of resistance to cancer therapy (Friedlaender et al., 2020). On the other hand, SCLC has a survival rate of between 15–20 months, because of its aggressiveness and invasiveness. SCLC develops early metastases, responds poorly to conventional treatment, and relapses in most cases (Zhong et al., 2020).

Epigenetic alterations are among the factors that drive tumor progression. In particular, cancer cells show an altered acetylation profile. This modified profile plays an essential role in tumor progression and poor response to lung cancer therapies (Zito Marino et al., 2019). Histone deacetylases (HDACs) are enzymes that regulate chromatin remodeling. HDACs remove acetyl groups from acetylated histones, which are essential to forming a scaffold for encasing and condensing DNA in the cell nucleus. Of note, the term acetylation may also refer to other types of protein acetylation; for example, that occurring in the O-linked acetylation of threonine and serine (Narita et al., 2019). The present review alludes only to the acetylation occurring in the amino group located on the epsilon carbon of the lysine side chain.

HDACs not only can regulate the function of histones but also non-histone proteins. While histones support compaction and chromatin remodeling, non-histone proteins take part in the regulation of DNA replication and RNA synthesis. HDACs have multiple biological functions in health and disease because they regulate various cellular processes such as cell proliferation, cell cycle, survival, and apoptosis (Kelly and Cowley, 2013; Chen et al., 2015; Narita et al., 2019; Wang P. et al., 2020). HDACs are critical for the reactivation of epigenetically silenced tumor suppressor genes (Parbin et al., 2014). Cumulative evidence shows that aberrant expression or activation of HDACs promotes carcinogenesis and contributes to the development of resistance to lung cancer therapies (Damaskos et al., 2018). Hence, several groups have focused on the research of HDAC inhibitors, which have improved treatments against this deadly disease (Shanmugam et al., 2022).

Because HDACs are attractive targets for treating cancer, there has been a search for novel HDAC inhibitors like phytochemicals. Fruits, seeds, vegetables, and dietary supplements contain many phytochemicals. For example, curcumin, apigenin, EGCG, resveratrol, and quercetin are commonly found in the diet. These phytochemicals have several effects, such as being antioxidants, and chemopreventives and they inhibit tumor growth (To and Cho, 2021). Phytochemicals modulate the epigenome through various mechanisms including the lysine acetylation of histone and non-histone proteins. Also, the combination of standard treatments and certain phytochemicals has been shown to restore sensitivity to lung cancer therapies (Wright et al., 2017; Gao and Tollefsbol, 2018).

In this review, we describe recent advances in HDACs and their inhibitors for the development of more effective cancer therapies. Furthermore, we describe some regulatory mechanisms by HDACs, focusing on treatment resistance. In addition, we also review the role of cancer stem cells and related promoter cells of resistance in HDACs-mediated therapy. Finally, we discuss the role of phytochemicals as a possible therapy, which may be used in combination with standard treatment.

## 2 Classification and function of HDACs in lung cancer

Histone deacetylases (HDACs) regulate chromatin remodeling by catalyzing the removal of acetyl groups from lysine residues in the histones. This process occurs when the chromatin is highly packed and is known as the heterochromatin state. This condition inhibits gene transcription (Li G. et al., 2020). There are 18 human HDACs enzymes, which are classified into four classes: Class I HDACs (HDACs 1, 2, 3, and 8); Class II, divided into two subgroups IIa (HDAC4, -5, -7, and -9) and IIb (HDAC-6 and -10); Class III, known as Sirtuins (SIRTs), consisting of SIRT 1-7 and Class IV with only HDAC-11 (Hu et al., 2000; Seto and Yoshida, 2014). Class I HDACs are commonly located in the nucleus, except for HDAC-3, which translocates to the cytoplasm. Class IIa and IIb HDACs can shuttle between the nucleus and cytoplasm. Class III SIRT 3, 4, and 5 localize in the mitochondria. SIRT6 and SIRT7 locate in the nucleus, and SIRT1 and 2 localize in the nucleus and cytoplasm. Class IV is only present in the cell nucleus (Schlumm et al., 2013; Guo et al., 2015; Chen et al., 2020).

Human HDACs are classified into two families based on their activity and structural homology. The zinc-dependent family consists of class I, IIa, IIb, and IV HDACs. Class, I HDACs -1, -2, -3, -4, -5, -7, and -8 have a catalytic domain and a nuclear localization signal (NLS) (Schuetz et al., 2008). Class IIa HDACs also have myocyte enhancer factor 2 (MEF2) and chaperone binding motifs (McKinsey et al., 2001). Class IIb HDAC-6 and -10 have leucine-rich motif-binding and ZnF-binding motifs (Tong et al., 2002; Milazzo et al., 2020). Class I HDAC-1 and HDAC-2 can form homo- and heterodimers whose catalytic domains or active cores localize within the deacetylase complex, which is required for protein deacetylation. The HDAC-1/HDAC-2 heterodimer recruits transcription factors, such as Sp1, Sp3, p53, NF-B, and YY1. This heterodimer binds to DNA through the formation of multiprotein corepressor complexes. Among the components are Sin3A, nucleosome remodeling deacetylase (NuRD), DNA -repressor of repressor element-1 silencing transcription factor (CoREST), and mitotic deacetylase (MiDAC). These complexes mediate HDAC-1/HDAC-2 phosphorylation and regulate cellular activation (Doetzlhofer et al., 1999; Grimes et al., 2000; Adams et al., 2018; Lee et al., 2021). The second HDAC family includes class III HDACs (SIRT1–7). Sirtuins require the NAD+ cofactor to be active and are structurally unrelated to the HDACs above mentioned (Borra et al., 2004; Spiegel et al., 2012).

**FIGURE 1:**
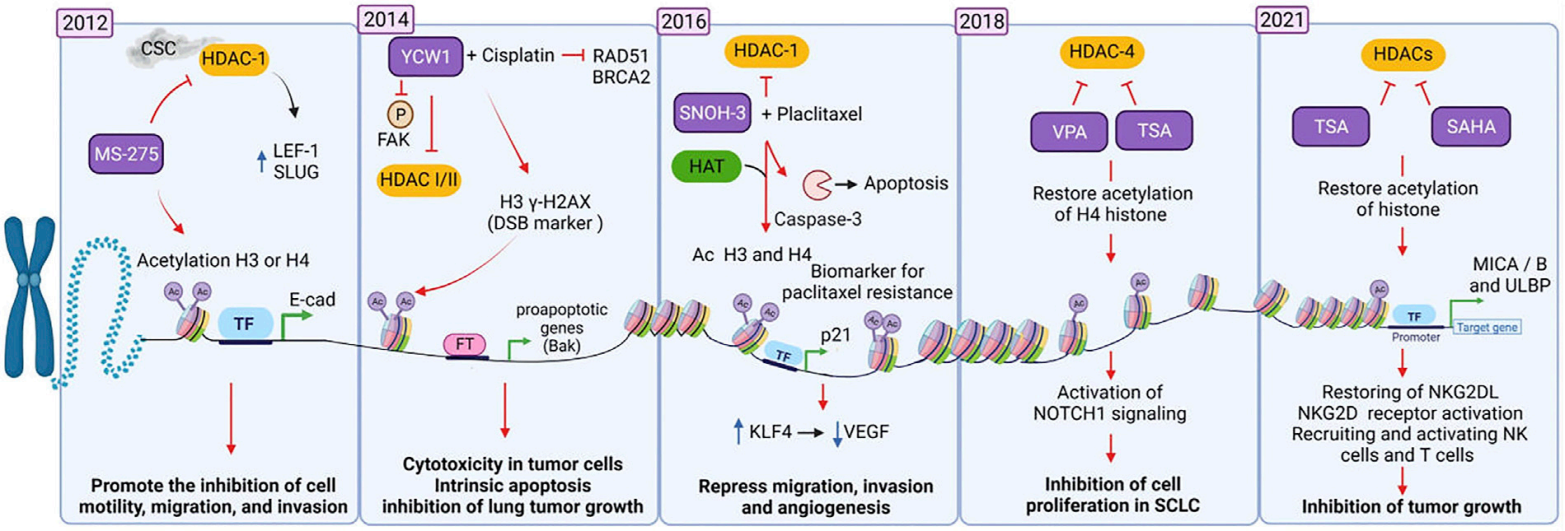
The different states of chromatin are epigenetically regulated by HDACs and HDAC inhibitors, which determine the transcriptional activation or silencing of genes involved in lung cancer development. Studies from 2012 to 2021 showing the role of HDACs and HDAC inhibitors in the development of therapies against lung cancer. Created with biorender.com CSC, cigarette smoke condensate; iHDACs (MS-275, YCW1, SNOH-3, VPA = Valproic acid, TSA = Trichostatin A, SAHA = Vorinostat), Ac, acetylation. 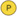
 Phosphorylation 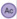
Acetylation 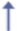
Up-regulation 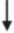
Activation 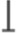
Inhibition 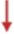
Mechanism promoted by treatments

Over the past decade, several groups have addressed the study of the structure and mechanisms of remodeling of HDACs. This research has led to the characterization of HDAC inhibitors, which are candidates for the development of more efficient anti-lung cancer therapies (see Figure 1). Here we describe some of these investigations chronologically to show how rapidly this field has developed. For starters, Nagathihalli et al. (2012) found that heavy smokers may develop a mesenchymal phenotype in lung cancer associated, which is associated with poor patient survival. *In vitro* analyzes showed that cigarette smoke condensate (CSC) promotes the depletion of epithelial markers such as E-cadherin and upregulates mesenchymal markers in lung cancer cell lines. HDAC-1 mediates the loss of E-cadherin expression by CSC, which increases the expression of T-cell factor -1 (LEF-1) and Slug. Conversely, the inhibitor of HDACs Entinostat (MS-275) restores the expression of E-cadherin and inhibits cell motility, migration, and invasion by acetylating histones H3 and H4 (Nagathihalli et al., 2012). Huang et al. reported in 2014 that the YCW1 inhibitor suppresses class I and II HDACs expression *in vitro* in lung cancer cells by acetylating histones H3 and H4 and tubulin (a non-histone protein). YCW1 induces cytotoxicity by activating the mitochondrial apoptotic pathway by overexpressing Bak and decreasing Bcl-XL protein expression. YCW1 suppresses invasion and metastasis through dephosphorylation of focal adhesion kinase (FAK). Also, the combination of YCW1 with cisplatin inhibits lung tumor growth in xenograft models due to a synergistic effect (Huang et al., 2014).

In patients with advanced lung cancer who have demonstrated resistance to paclitaxel treatment, tumor biopsies show high levels of HDAC-1 and a loss of p21 expression, which correlates with acquired resistance to treatment (Wang et al., 2016a). A study, published in 2016, showed that the inhibitor SNOH-3 ((2E)-N-hydroxy-3-{4-[(3-trifluoromethylphenylamino) sulfonyl] phenyl}-2-propenamide) inhibits the activity of HDAC-1 and restores sensitivity to paclitaxel in resistant lung tumor cells. The mechanism is based on the acetylation of histones H3 and H4 and the increased expression of the p21 molecule. SNOH-3 alone or in combination with paclitaxel promotes apoptosis by cleaving caspase-3 and PARP (poly-ADP-ribose polymerase 1) and decreases the expression of the anti-apoptotic proteins XIAP and survivin. SNOH-3 suppresses migration, invasion, and angiogenesis by inducing Kruppel Like Factor 4 (KLF4) expression, thus decreasing VEGF expression (Wang L. et al., 2016).

Sun et al. reported in 2018 that activation of Notch inhibits cell proliferation by activating the Notch fragments (ICN1, ICN2, ICN3, and ICN4) in SCLC. The authors found that valproic acid (VPA) and trichostatin A (TSA) decrease the expression of HDAC-4, restoring histone H4 acetylation. This in turn promotes Notch1 signaling and induces higher expression of somatostatin receptor 2 (SSTR2) and the Notch-directed genes HES1 and p21 (Sun et al., 2018). Besides, Zhu et al. showed that TSA and vorinostat (suberoylanilide hydroxamic acid, SAHA), both HDACs inhibitors, promote the expression of MICA/B, leading to activation of NK cells and tumor growth inhibition (Zhu et al., 2021).

Studies made in the last 5 years (2017–2021) have characterized HDAC inhibitors in lung cancer, using *in vitro* and *in vivo* assays. These studies have reported that HDAC inhibitors act against class I, II, and III HDACs. These HDACs act at several levels; for instance, they inhibit tumor cell proliferation and tumor growth. HDACs also sensitize to therapy against lung cancer. Currently, clinical trials for HDAC inhibitors in lung cancer are in phases I and II (See Table 1).

**TABLE 1:** HDACs regulate the resistance to therapies in lung cancer.

Class HDAC, I, IIa, IIb and III	Biological Function/cancer hallmarks clinical impact	Target/signaling pathways	Resistance/sensitivity therapies	HDACs inhibitors promote sensitivity	*In vitro*, *in vivo* and clinical trials	Reference
HDAC-1	Cell proliferation and migration,	HDAC/RXR/HtrA1	Cisplatin resistance	Panobinostat (LBH-589), Vorinostat (SAHA), CG347B	A549, H460, H1299, Balb/c-nu mice	Wang et al. (2020b)
HDAC-1	tumor progression, poor survival	CHK1, E-cadherin	Erlotinib resistance	Panobinostat	Clinical phase 1, (EGFR-mutant NSCLC patients)	Gray et al. (2014)
HDAC-1, -2, -4	Epithelial-to-mesenchymal transition (EMT)	CREBBP, CDH1		Pracinostat	H1882, DMS53, 293FT, Athymic Foxn1nu mice	Jia et al. (2018)
HDAC-1	Apoptosis inhibition	BAX-Ku70 interaction	Amphiregulin Promotes Resistance to Gefitinib	TSA, SAHA	H358 and H322, NMRI nude mice	Busser et al. (2010)
HDAC-1	Apoptosis inhibition, poor prognosis, tumor recurrence	C/EBP-β/TRIB1/HDAC/p53 axis	Cisplatin, doxorubicin resistance	SAHA	H460, A549 and H1299, xenograft model mice	Wang et al. (2017)
HDAC-1	Apoptosis inhibition	BIM	Gefitinib and erlotinib resistance	SAHA	PC-3, PC-9, HCC827, and HCC2279, BALB/cAJcl-nu/nu mice	Nakagawa et al. (2013)
HDAC-1, -2, –3	Apoptosis inhibition, promote cell cycle	p21-cyclin B1	Gefitinib resistance	TSA, SAHA + combination with Silibinin	H1299, H358, and H322, athymic (nu/nu) nude mice	Mateen et al. (2012)
HDAC-1, -2, –3	Apoptosis inhibition	FLIP	Cisplatin-resistant	SAHA, LBH-589	H460, A549 and 34LU	Riley et al. (2013)
HDAC-6	Inhibited proliferation, induce apoptosis	EGFR	Gefitinib resistance	CAY10603	A549, HCC827 and H1975	Wang et al. (2016b)
HDAC-1, -2, -3	Radiosensitization, induce apoptosis	FLIP and caspase-8	Resistance to Radiation	SAHA	A549, H460, H1373 CCD34Lu and HCC15	McLaughlin et al. (2016)
HDAC-1, -3, -6, -8	Apoptosis inhibition cell proliferation migration, invasión,	p21	Paclitaxel resistance	SAHA, SNOH-3	A549 and NCI-H1299, and HUVEC, SCID mice	Wang et al. (2016a)
HDAC-1	Growth inhibition, induce apoptosis	EGFR	Erlotinib resistance	SAHA, MPT0E028	CL97, A549, H1975, and H1299, nude-athymic mice	Chen et al. (2013)
HDAC-1, -2	DNA repair inhibition, promote growth size tumor	ACTL6A	Cisplatin resistance	Panobinostat	A549, H1299, NOD scid IL2 receptor g chain knockout (NSG) mice	Xiao et al. (2021)
HDAC-1	Poor prognosis, induced tumor cell growth	HDAC/OAZ1 axis	Cisplatin resistance	S11	A549, NCI-H460, and NCI-H1299, BALB/c nude mice	Sun et al. (2019)
HDAC-1	EMT	ALK	Crizotinib, alectinib, lorlatinib and ceritinib resiistance	SAHA	PF240-PE, PF240, PF240-PC, PF521	Stockhammer et al. (2020)
HDAC-1	Apoptosis inhibition, cell proliferation	PTEN	Erlotinib resistance	SAHA	PC-9, PC-9/ER, H1975	Wu et al. (2020)
HDAC-3	Apoptosis inhibition	BIM	Osimertinib resistance	SAHA	PC-9 and PC-3, BALB/c-nu/nu mice	Tanimoto et al. (2017)
Pan-HDAC	Cell proliferation, apoptosis inhibition	EGFR	Carboplatin resistance	Panobinostat	A549, Calu-1, H226, H460, H838 and SKMES-1, NOD-SCID xenograft mice	Wang et al. (2018b)
HDAC-1	Apoptosis inhibition and autophagy	EGFR	Gefitinib and erlotinib resistance	SAHA	PC-9, H1975, athymic nude mice	Lee et al. (2015)
HDAC-1, -2	Promotecell growth and cell proliferation, apoptosis inhibition	EGFR	Erlotinib resistance	YF454A	A549, H1299, H1975, PC9, HCC827, BALB/cA nude mice	Yu et al. (2017)
HDAC-1, -4	Induced cell proliferation, apoptosis inhibition	HIF-1α	Cisplatin resistant	panobinostat	NCI-H23, A549	Fischer et al. (2015)
HDAC I/IV	Inhibit an efective antigen-specifc immune response	PDL-1	PDL-1 immunotherapy	Mocetinostat is a spectrum-selective inhibitor	NCI-H23, NCI-H1299, NCI-H1437, NCIH1703, NCI-H1792, NCI-H1838, NCI-H2122 and CT26. WT (CT26).	Briere et al. (2018)
HDAC-2	Promote resistance to therapy	ABCA1	Cisplatin resistant	Valproic acid (VPA)	A549, H358	Chen et al. (2017b)
HDAC-1, -2, -3	Induced cell cycle and decreased apoptotic	Inhibit the PI3K/AKT and RAS/MAPK pathways	Icotinib resistant	chidamide	A549, HCC827, HCC827IR, Bagg Albino (BALB/c) athymic nude mice	Zhang et al. (2019)
HDAC	Promote Cell cycle apoptosis inhibition, decreased DNA damage repair activity	FoxM1 and MYC	Olaparib resistant	CUDC-907	DMS273, H82, H526, H69, and H446, patient-derived xenografts (PDXs) model	Ma et al. (2021)
HDAC-1,- 2,- 3, -6,	Promotion of tumor growth	BET	Osimertinib resistance	SAHA, TSA	H1975 and HCC827, athymic (nu/nu) mice	Meng et al. (2021)
SIRT1	Induces cell cycle, proliferation, and apoptosis is suppressed in cisplatin-resistant cells.	p53, p21,	Cisplatin resistant		A549 and A549/CADD cells,	Yu et al. (2020)
SIRT1	Induced apoptosis resistance and chemoresistance	β-TrCP- XRCC1	Cisplatin resistant	EX-527	H460	Yousafzai et al. (2019)
SIRT5	Regulates drug resistance, poor overall- and disease-free survival	Nrf2	Cisplatin resistant		A549, LU99 and NCI-H460, nu/nu mice	Lu et al. (2014)

Refs (Busser et al., 2010; Mateen et al., 2012; Chen et al., 2013; Nakagawa et al., 2013; Riley et al., 2013; Gray et al., 2014; Lu et al., 2014; Fischer et al., 2015; Lee et al., 2015; Wang L. et al., 2016; Wang et al., 2016b; McLaughlin et al., 2016; Chen J. H. et al., 2017; Tanimoto et al., 2017; Wang et al., 2017; Yu et al., 2017; Briere et al., 2018; Wang L. et al., 2018; Jia et al., 2018; Wei et al., 2018; Sun et al., 2019; Yousafzai et al., 2019; Zhang et al., 2019; Wang W. et al., 2020; Stockhammer et al., 2020; Wu et al., 2020; Yu et al., 2020; Ma et al., 2021; Meng et al., 2021; Xiao et al., 2021).

On the other hand, posttranslational modifications of non-histone proteins are involved in the tumorigenesis of lung cancer. As components of epigenetic regulatory mechanisms, HDACs may also target non-histone proteins involved in several cellular processes (e.g., migration, invasion, and metastasis) (Harada et al., 2016). For instance, S100A6 (S100 Calcium Binding Protein A6) promotes cell proliferation and inhibits cell death in lung cancer through the deacetylation of p53; thus, p53 is inactivated (Li et al., 2019).

Modulation of non-histone proteins using HDACs may help against cancer. For example, quisinostat is an inhibitor of HDAC-6 in lung cancer. Quisinostat promotes high acetylation on H3 and H4 histones, as well as α-tubulin, a non-histone protein. This inhibitor increases the production of reactive oxygen species (ROS), promotes loss of mitochondrial membrane potential (ΔΨm), and induces apoptosis through an imbalance between pro- and anti-apoptotic proteins of the Bcl-2 family. Quisinostat also induces the activation of the p53 pathway by acetylation at K382/K373 sites, which increases the expression of p21(Waf1/Cip1), p27, p57, and cyclin D1 repression. All these events lead to cell cycle arrest in the transition of G1-to S-phases. Quisinostat inhibits cell migration and metastasis by suppressing the EMT phenotype, although the mechanism is not very clear (Bao et al., 2016).

Another report showed for the first time that HADC-7 has a function as an oncogene in lung cancer. Forty-four percent of lung cancer tumors exhibit high HDAC-7 expression, which correlates with poor prognosis in patients. High expression of HDAC-7 inactivates the non-histone protein STAT3 through its deacetylation. This promotes cell proliferation and inhibits apoptosis in lung cancer (Lei et al., 2017). Another study showed that acetylation of lysine residues at K185 and K201 sites of the C1 member of the aldo-keto reductase 1 family (AKR1C1) induces a pro-metastatic phenotype in NSCLC cells both *in vivo* and *in vitro*. The acetylation of AKR1C1 activates STAT3, which promotes metastasis. However, the physical interaction between SIRT2-and AKR1C1 represses the metastatic phenotype through deacetylation of AKR1C1. This process inhibits STAT3 expression (Zhu et al., 2021).

Tyrosine kinase inhibitors (TKIs) are commonly used to treat lung cancer. One of the targets for which they have been designed is anaplastic lymphoma kinase (ALK). ALK is a tyrosine kinase originally described in lymphoma that is aberrantly expressed in several tumors. In NSCLC, chromosomal rearrangements involving the ALK gene loci on chromosome 2 are in approximately 3–5 percent of NSCLC tumors. Although lung tumors harbor ALK rearrangements and may be sensitive to TKIs, they develop several mechanisms of resistance. These include the acquisition of a secondary mutation within the ALK tyrosine kinase domain, which is present in approximately one-third of resistant cases. The most common resistance mutation is the gatekeeper L1196M mutation, followed by the G1269A mutation. Other mutations occur at residues 1151, 1152, 1156, 1174, 1202, 1203, and 1206. The G1202R mutation confers resistance to crizotinib and second-generation ALK inhibitors (alectinib, brigatinib, ceritinib, and ensartinib) but is sensitive to lorlatinib (Katayama et al., 2012; Recondo et al., 2020; Takahashi et al., 2020).

The combination of HDACs with ALK inhibitors is an alternative for patients whose tumor develops resistance. Fukuda et al. (2019) showed that pretreatment with the HDAC inhibitor quisinostat downregulates the expression of miR-200c, leading to higher expression of ZEB1 and promoting tumor cell sensitivity to crizotinib. Stockhammer et al. (2020) found that the highly resistant PF240-PE tumor cells to crizotinib and alectinib became sensitive to these ALK inhibitors after treatment with vorinostat. Thus, combining therapies using TKIs and HDACs could be an option to increase the effectiveness, and response treatment to increase survival and disease-free survival in lung cancer patients.

## 3 Mechanisms of resistance to therapy regulated by HDACs in lung cancer

Despite advances in the development of new therapies against lung cancer, the development of resistance to treatment is a current problem that requires further research effort and investment. Several factors are implicated in tumor treatment resistance, including intratumoral heterogeneity, genetic instability, and phenotypic plasticity. Molecular and phenotypic changes within tumor cells favor clonal selection, leading to tumor progression. Genetic instability and tumor plasticity confer intrinsic resistance to therapy by inducing mutations, deletions, and amplifications. However, epigenetic changes also favor the development of resistance to treatments. In particular, the aberrant deacetylation of histone and non-histone proteins by HDACs is a relevant mechanism that is associated with developing therapy resistance in lung cancer.

**FIGURE 2:**
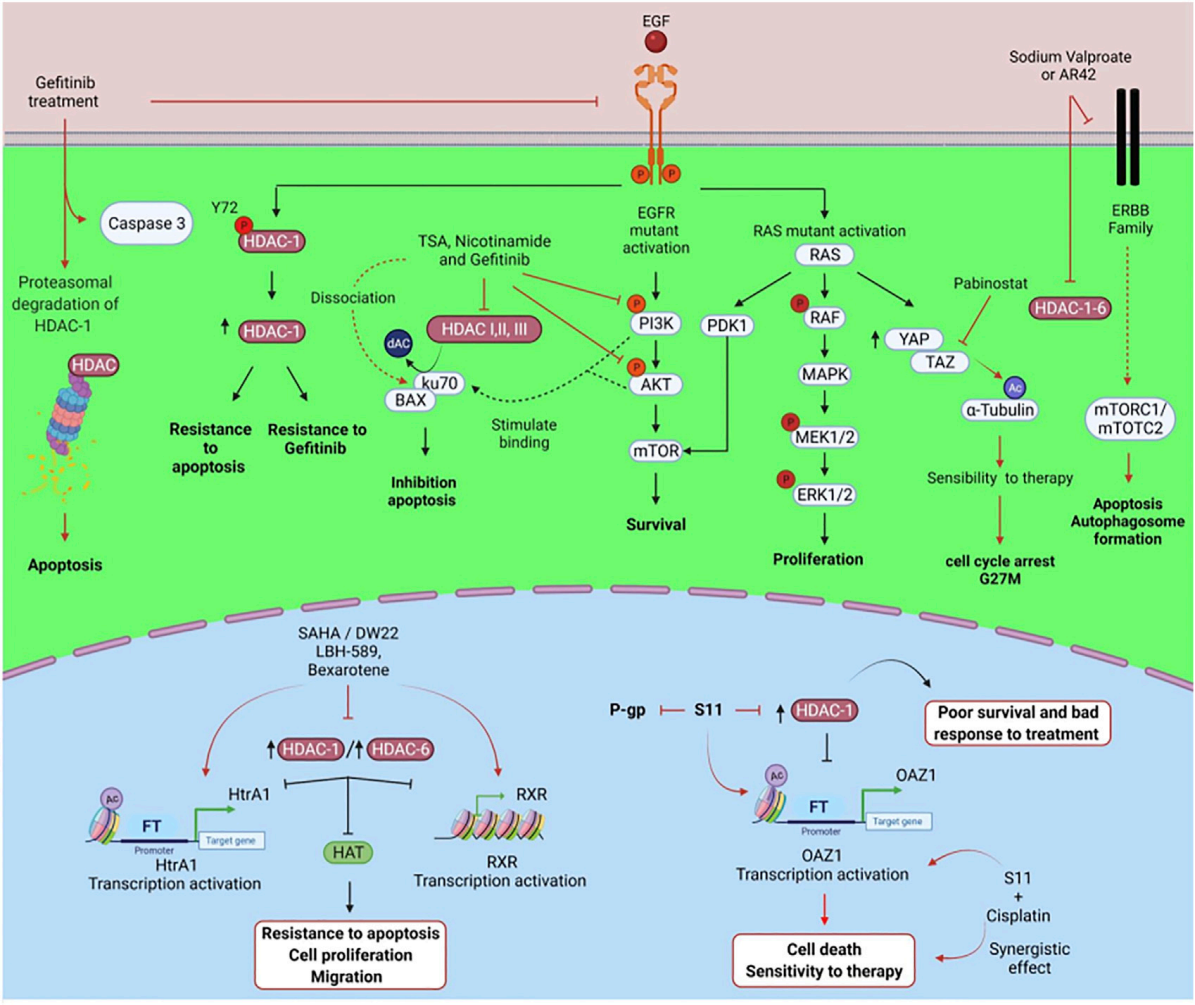
Mechanisms of regulation of HDACs and signaling pathways associated with resistance to therapies in lung cancer. Epigenetic therapies with HDAC inhibitors regulate different signaling pathways, proteasome activity, and transcriptional repression of genes involved in the cancer hallmarks of lung cancer. Created with Biorender.com. 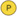
Phosphorylation 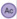
Acetylation 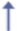
Up-regulation 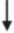
Activation 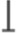
Inhibition 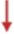
Mechanism promoted by treatments

Multiple signaling pathways play a part in HDAC-related drug resistance in lung cancer. Among them are epidermal growth factor receptor (EGFR)/HDAC-1, HDAC/retinoic X receptor (RXR)/high-temperature requirement factor A1 (HtrA), and HDAC/ornithine decarboxylase antizyme 1 (OAZ1) (Table 1 and Figure 2). EGFR is a transmembrane receptor tyrosine kinase whose overexpression is linked to the development of lung cancer. Most patients having EGFR-positive lung tumors are successfully treated with TKIs as elective therapy. However, some cases develop EGFR mutations that confer acquired resistance to standard therapy and TKIs (Nagano et al., 2018).

To target this phenomenon, Wei et al. (2018) treated with vorinostat lung cancer cells harboring EGFR mutations associated with TKI resistance. Vorinostat suppresses cell tumor cell growth by dephosphorylating ERK and AKT. Vorinostat also induces cell cycle arrest and apoptosis and represses VEGF expression, which results in reduced angiogenesis.

EGFR promotes the phosphorylation of HDAC-1, an event required for its stability and increased expression. Phosphorylation of the Tyr72 site on HDAC-1 promotes the antiapoptotic function of HDAC-1 and resistance to gefitinib in lung adenocarcinoma (Bahl et al., 2021). BCL-2–interacting mediator of cell death (BIM) is a BH3-only proapoptotic member of the Bcl-2 protein family that induces apoptosis. Upregulation of BIM induces apoptosis in EGFR-mutated forms of NSCLC through EGFR-TKIs. BIM deletion polymorphism is associated with resistance to EGFR-TKIs, such as gefitinib and erlotinib. This polymorphism confers an inherent TKIs-resistant phenotype (Ng et al., 2012; Nakagawa et al., 2013).

Nakagawa et al. (2013) found that vorinostat increases BIM expression, which restores sensitivity to gefitinib in EGFR-TKI-resistant cells. In xenograft models, the combination of gefitinib with vorinostat markedly reverts the growth of tumors bearing the BIM polymorphism compared to gefitinib alone. Similarly, Tanimoto et al. (2017) found that vorinostat combined with osimertinib reverses the resistance of EGFR-mutated NSCLC cell lines presenting the BIM deletion polymorphism. Vorinostat affects the alternative splicing of BIM mRNA by increasing the expression of active BIM protein and also inhibits HDAC-3. In xenograft models, combining vorinostat with osimertinib reverts tumors in EGFR-mutated NSCLC cells that are homozygous for the BIM deletion polymorphism.

Mesenchymal-epithelial transition factor (cMet) is a transmembrane tyrosine kinase receptor with pleiotropic functions. Phosphorylation for a hepatocyte growth factor (HGF) activates cMet. Elevated HGF levels and c-Met overexpression are associated with poor prognosis in lung cancer. MET gene amplification is a known mechanism of resistance to TKIs (Mo and Liu, 2017; Liang and Wang, 2020). He et al. explored the antitumor activity of GCJ-490A. This is a novel pan-HDAC inhibitor that exerts potent inhibitory activity against HDAC-1, HDAC-3, and HDAC-6. The authors studied the effect of GCJ-490A alone or in combination with the EGFR inhibitor gefitinib against NSCLC. They found that GCJ-490A inhibits NSCLC cell proliferation and induces apoptosis *in vitro* and *in vivo*. The mechanism is *via* inhibition of HDAC-1 and HDAC-6, increasing histone acetylation of the IKK promoter. This in turn enhances IKK transcription, which increases c-Met expression (He et al., 2022).

Mutations in Kirsten rat sarcoma viral oncogene (KRAS) in NSCLC are associated with poor prognosis and resistance to TKIs. KRAS mutations induce resistance to gefitinib. This is due to the high expression of amphiregulin (AREG) and the insulin-like growth factor-1 receptor (IGF1R), which activates the PI3K/Akt pathway. Lung adenocarcinomas harbor mutations in the KRAS oncogene, and 80% of these mutations occur in codon 12. KRAS mutations are independent of EGFR mutations (Salgia et al., 2021). Sustained activation of PI3K/AKT stimulates Ku70 binding to BAX, leading to apoptosis inhibition and promoting cell proliferation. The mechanism is through class I/II and III/sirtuin HDACs, which deacetylate Ku70. Jeannot et al. (2014) investigated the role of acetylation in controlling the interaction between BAX and Ku70. They also studied how the acetylation of Ku70 participates in the PI3K/AKT pathway and gefitinib resistance. The authors found that TSA (a class I/II HDAC inhibitor) and nicotinamide (a class III/sirtuin deacetylase inhibitor) sensitize H358 cells to gefitinib-induced apoptosis. The combination of TSA and nicotinamide induces apoptosis and significantly sensitizes cells to gefitinib compared to gefitinib treatment with TSA or nicotinamide alone. TSA and nicotinamide inhibit gefitinib-induced activation of p-AKT. The mechanism is through an additive effect via activation of caspase 3 and dissociation of BAX/Ku70 by acetylation of Ku70.

The Hippo signaling pathway is a kinase cascade containing a Yes-associated protein (YAP) and a transcription coactivator with a PDZ-binding motif (TAZ). Hippo, YAP, and TAZ play a role in several processes such as early airway bifurcation morphogenesis, epithelial lineage differentiation, cellular transition to air respiration, injury repair, and tissue regeneration. Nevertheless, these molecules are also involved in carcinogenesis and cancer progression (Warburton, 2020). Lung cancer cells with mutated KRAS express high protein levels of YAP/TAZ. TAZ is a transcriptional activator that regulates organ size but is also involved in tumor growth and migration. TAZ promotes the secretion of AREG, which activates the EGFR signaling pathway (Yang et al., 2012; Chao et al., 2021). Gefitinib and panobinostat may be effective against gefitinib-resistant lung cancer cells that exhibit mutations in KRAS and EGFR. Panobinostat induces TAZ repression, which leads to cell death by cleavage of PARP, caspase-3, and caspase-9. Increasing histone H3 acetylation of the non-histone protein tubulin promotes tumor cell sensitivity to gefitinib (Lee et al., 2017). *In vitro* and *in vivo* studies show that the combination of resminostat (an HDAC-6 inhibitor) with docetaxel induces microtubule stabilization through the polymerization and acetylation of H3-histone into tubulin in lung cancer. Microtubule stabilization arrests cells in the G2/M phase of the cell cycle, suppresses cell proliferation and causes cell death mediated by activation of caspases -3 and -7 (Konishi et al., 2017).

One of the most widely used therapies for NSCLC patients with mutated EGFR is afatinib (Wu et al., 2021). Yet, when patients develop resistance to afatinib, there are few options left to treat these patients. A study using H1975 lung cancer cells resistant to afatinib showed that treatment with HDAC inhibitors sodium valproate or AR42 decreased the expression of several members of the tyrosine kinase receptor family: ERBB1, ERBB2, ERBB3 and ERBB4, and c-MET. These HDAC inhibitors also decrease the expression of HDAC-1, HDAC-2, HDAC-3, HDAC-4, HDAC-6, and HDAC-10. The combination of neratinib with sodium valproate inactivates mTORC1 and mTORC2, which induces apoptosis and autophagosome formation. Besides, this combination reduces the expression of PD-L1, PD-L2, and ornithine decarboxylase (ODC). These molecules are implicated in poor response to treatment. The combination of neratinib and sodium valproate increases the expression of MHC-A molecules (Booth et al., 2019).

The serine peptidase HtrA1 participates in cisplatin (CDDP) resistance by promoting a cancer stem cell phenotype in lung cancer (Li et al., 2020b). Due to the high expression of HDAC-1 and HDAC-6 in cancer cells, HtrA1 expression is low, as well as the expression of the retinoid X receptor (RXR). This results in the inactivation of acetyltransferase and increased resistance to CDDP. The combination of HDAC inhibitor panobinostat (LBH-589) with the RXR agonist bexarotene (Bexa) has a synergistic effect that increases HtrA1 and RXR expression and inhibits HDAC-1 and HDAC-6. Thus, HDACs and RXR play a co-regulatory role in HtrA1 expression. Another mechanism of HtrA1 regulation is through DW22, which inhibits HDACs and activates RXR, resulting in increased transcription of HtrA1. DW22 and vorinostat increase the ability of RXR to bind to the promoter-specific sites of HtrA1, and acetylated H4 and H3 bind to the HrtA1 promoter. All this reduces tumor cell invasion and migration and suppresses tumor growth *in vivo* (Wang W. et al., 2020).

P-glycoprotein (P-GP), a drug efflux pump, is overexpressed in multiple cancers and is associated with multidrug resistance (Seelig, 2020). Sun et al. (2019) reported that 60% of tumor tissues, obtained from patients treated with CDDP, show decreased ornithine decarboxylase antizyme 1 (OAZ1) expression and high HDAC-1 expression. This correlates with poor survival and poor treatment response. The authors also found that the HDAC inhibitor S11 suppresses P-gp and HDAC-1 and increases the expression of OAZ1. S11 increases the accumulation of acetylated H4 in the OAZ1 promoter region. Besides, S11 decreases cell migration and colony formation. The combination of S11 with CDDP is synergistic and promotes cell death *in vitro* and *in vivo*. This results in an increased sensitivity to CDDP therapy (Sun et al., 2019).

## 4 Cancer stem cells promote therapy resistance through HDACS in lung cancer

Tumor heterogeneity contributes to progression, poor response to treatment, and development of acquired resistance to therapies in solid tumors. Tumor heterogeneity comprises intertumoral and intratumoral heterogeneity. Intertumoral heterogeneity consists of genetic variations among patients with the same type of tumor. While intratumoral heterogeneity is conformed by different cancer cells containing genetic variations and epigenetic changes, as well as changes in the regulation of gene expression (Brady et al., 2021). Solid tumors are composed of cancer cells, cancer stem cells (CSCs), and stromal cells such as endothelial cells, tumor-associated fibroblasts, mesenchymal stem cells, and tumor-associated macrophages (TAMs). All these cells form part of a complex architecture supported by blood vessels and the extracellular matrix, which all in all comprise the tumor microenvironment. Besides, soluble factors such as nutrients, growth factors, and cytokines, as well as the oxygen supply are essential components of the tumor microenvironment. The interaction of all these factors contributes to the development of angiogenesis, cell proliferation, invasion, and metastasis in lung cancer (Hass et al., 2020).

**FIGURE 3:**
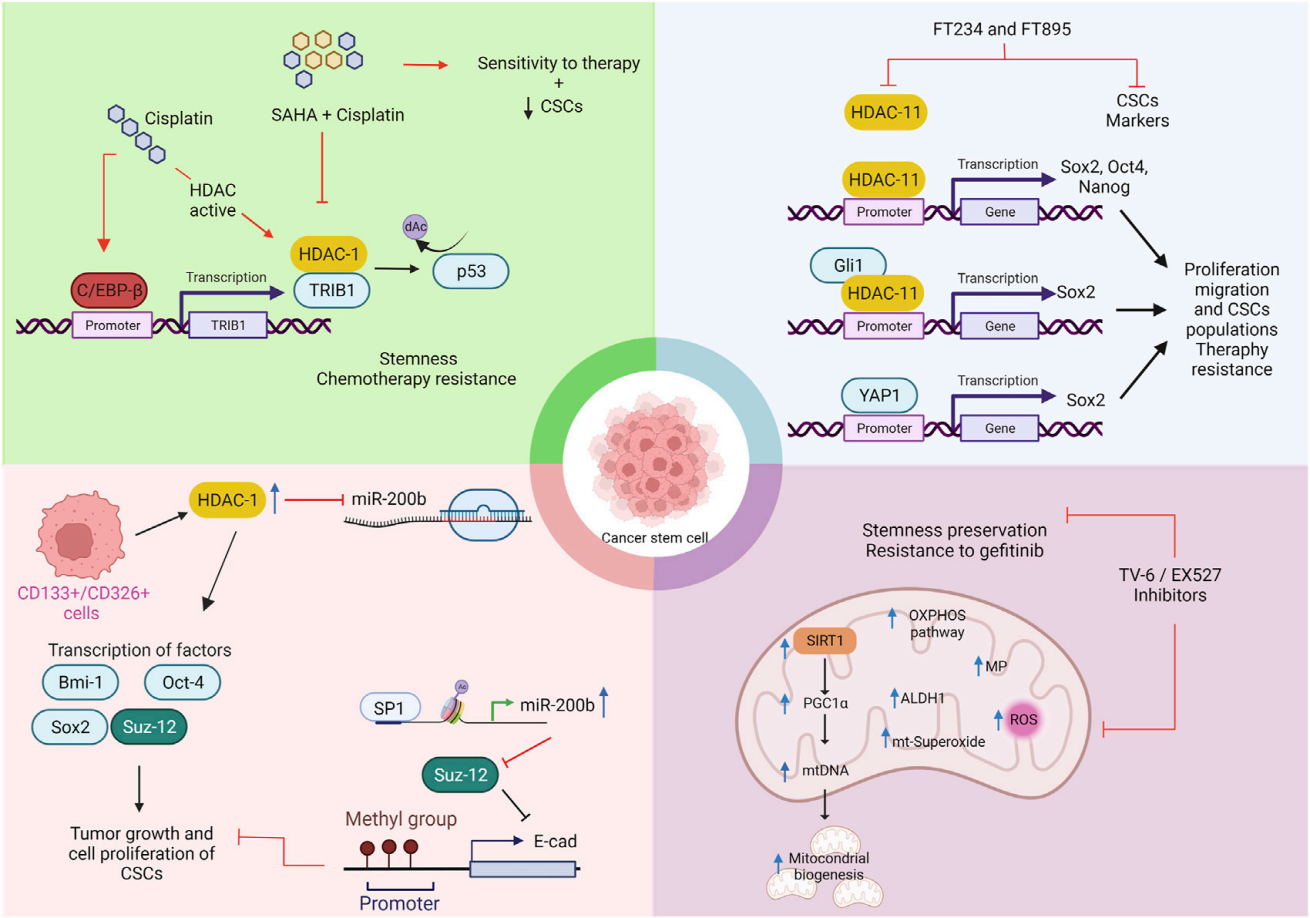
Strategies of CSCs subpopulations that promote the phenotype resistant tumor cells to therapies mediated by HDACs in lung cancer. Created with biorender.com HDAC inhibitors (SAHA = Vorinostat, FT234, FT895); Ac, acetylation; TRIB1 = tribbles pseudokinase 1; C/EBP-*β*, CCAAT enhancer binding protein beta; YAP1, yes associated protein 1; SOX2, SRY-Box Transcription Factor 2; OCT4, Octamer-binding transcription factor 4; Bim-1, BMI1 Proto-Oncogene, Polycomb Ring Finger; PRC2, polycomb repressive complex 2; Suz-12 = SUZ12 Polycomb Repressive Complex 2 Subunit; EED, embryonic ectoderm development; RbAP 46/48, retinoblastomabinding protein p48; EZH2, Enhancer Of Zeste 2 Polycomb Repressive Complex 2 Subunit; OXPHOS, oxidative phosphorylation; ROS, reactive oxygen species. 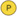
Phosphorylation 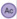
Acetylation 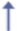
Up-regulation 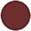
Methylation 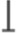
Inhibition 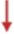
Mechanism promoted by treatments 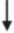
Activation

CSCs contribute to tumor progression, relapse, poor survival, and treatment resistance in lung cancer patients (see Figure 3) (71). CSCs are a small subpopulation of cancer cells that exhibit many genetic and epigenetic alterations. CSCs share properties with normal stem cells, such as the expression of transcription factors Nanog, Oct4, and Sox2, surface markers ALDH, and CD133. They also share the capacity for self-renewal and differentiation to multiple lineages (Saito, 2014).

Wang et al. reported that cisplatin, but not paclitaxel and doxorubicin, induces the enrichment of CSCs and confers multidrug resistance to NSCLC cell lines. Cisplatin-resistant tumors increase the expression of CSC transcription factors in a xenograft lung cancer model. Treatment with cisplatin promotes the interaction of HDAC-1 with pseudo kinase tribbles homolog 1 (TRIB1), resulting in the inactivation of p53 through its deacetylation (Wang et al., 2017). Even so, the combination of vorinostat and cisplatin enhances antitumor effects against NSCLC cell lines in a TRIB1-dependent manner. This combination also shrinks xenograft tumors (Eyers et al., 2017; Jadhav and Bauer, 2019).

Several studies showed that microRNAs regulate self-renewal, tumorigenicity, metastasis, and chemoresistance of CSCs in various human cancers. Chen et al. (2014) investigated whether miR-200b regulates CSCs derived from docetaxel-resistant lung adenocarcinoma cells. Results showed that CSCs derived from docetaxel-resistant lung adenocarcinoma cells downregulate miR-200b. Overexpression of HDAC1 represses miR-200b in CSCs through a specificity protein (Sp) 1-dependent mechanism. Upregulation of miR-200b reverses the chemoresistance of docetaxel-resistant adenocarcinoma cells by inducing cell cycle arrest and apoptosis. HDAC1 repression restores miR-200b, inhibits the enrichment of CSCs, and reverses the chemoresistance of CSCs. The mechanism is by regulating Suz-12, a miR-200b target, through the E-cadherin signaling pathway. These data suggest that HDAC-1/miR-200b/Suz-12-E-cadherin signaling regulates the formation of CSCs in docetaxel-resistant lung adenocarcinoma cells.

Bora-Singhal et al. (2020) found high levels of HDAC-11 in lung adenocarcinoma and squamous cell carcinoma tumor tissue compared to normal lung tissue. High levels of HDAC-11 correlate with poor treatment outcomes. HDAC-11 is upregulated in the cancer stem-like population (SP) from NSCLC cell lines (A549 and H1650). Elimination of HDAC-11 reduces self-renewal of cancer SP cells and decreases Sox2 expression, which is essential for maintaining this cell subset. HDAC-11 regulates Sox2 expression through interaction with the transcription factor Gli1. Highly selective HDAC-11 inhibitors FT234 and FT895 efficiently ablate the growth of drug-insensitive stem-like cells and therapy-resistant lung cancer cells. Treatment with synthetic inhibitors against HDAC-11 suppresses Sox2, reduces cell proliferation and migration, and decreases the CSC subpopulation.

While HDAC class I and IV overexpression promotes the development of CSCs in lung cancer, HDAC-10 (class IIb) acts as a tumor suppressor by targeting CSCs (Li et al., 2020c). Li et al. reported that HDAC-10 might act as a putative tumor suppressor in mice carrying a spontaneously activated oncogenic KRAS allele. HDAC-10 deletion accelerates early-stage KRAS-driven lung adenocarcinoma, increases macrophage infiltration within the tumor microenvironment, and shortens the survival time in mice. The authors found an increased number of highly tumorigenic and strain-like lung adenocarcinoma cells in Hdac10-deleted tumors compared to Hdac10 wild-type tumors. Deletion of HDAC-10 in knock-out tumor cells induces higher expression of SOX9 and genes associated with the TGF pathway, indicating a possible mechanistic association (Li et al., 2020c).

On the other hand, there is little evidence for the role of class III HDACs or sirtuins (SIRT 1–7) in lung cancer therapy resistance. Sun et al. (2020) characterized drug-resistant lung adenocarcinoma cell lines after treatment with gefitinib. They identified a subpopulation of tumor cells that exhibit strain-like properties, mitochondria-specific metabolic features, and expression of SIRT1 as a survival benefit. Treatment with tigecycline, a mitochondrial DNA translation inhibitor, or tenovin-6 (TV-6), a SIRT1 inhibitor, inhibits their dependence on mitochondrial oxidative phosphorylation (mtOXPHOS) and sensitizes them to a more pronounced and long-lasting therapeutic TKI effect. Combined therapy with TV-6 and gefitinib, but not single-agent therapy, induces tumor regression in xenograft mouse models. Furthermore, increased expression of SIRT1 and mtOXPHOS proteins in tumor tissues of lung adenocarcinoma patients is associated with recurrence and poor prognosis.

## 5 Clinical assays using HDACs for lung cancer treatment

We briefly describe the inhibitory activity of HDAC inhibitors against HDAC in assays *in vitro* in lung cancer. Different studies have reported the inhibitory activity against HDAC *in vitro*. For instance, SNOH-3 is a novel inhibitor of HDAC-1, HDAC-3, HDAC-6, and HDAC-8 isoforms, with half-maximal inhibitory concentration (IC50) of 46.5 ± 13.1, 28.1 ± 2.3, 42.3 ± 11.8 and 146.2 ± 52.6 nM, respectively. While vorinostat has IC50 values of 93.4 ± 16.9, 51.2 ± 9.2, 26.6 ± 6.3, and 195.9 ± 32.5 nM against these HDAC isoforms. The IC50 values were obtained from A549 and H1299 cell lines. SNOH-3 has better activity against HDAC-1 compared to HDAC-3, HDAC-6, and HDAC-8 in both cell lines. Similar results were observed with vorinostat. SNOH-3 promotes apoptosis through the scission of caspase-3 and PARP and decreases the anti-apoptotic proteins XIAP and survivin. Additionally, this inhibitor induces cell migration, invasion, and angiogenesis inhibition by mediating an increase in KLF4 expression and low VEGF expression (Wang L. et al., 2016).

Isoform selective HDAC inhibition can be one of the therapeutic strategies in the treatment of lung cancer. HDAC inhibitors have different IC50 values, as observed *in vitro* assays on lung cancer cell lines. Yet most HDAC inhibitors show IC50 values ranging from 4.5 to 10.5 mM concentrations. For example, panobinostat ranges between 4 and 31 nM, vorinostat ranges from >10 to 1975 nM, and mocetinostat has an IC50 value of 1000 nM. These inhibitors have a selective affinity to HDAC isozymes dependent on zinc (HDAC class I, IIa, IIb, and IV). Of note, all of them have completed clinical trials (Table 2). Only the CUDC-907 inhibitor has an IC50 ranging from 0.49–8.8 nM. Thus, CUDC-907 could be a potential candidate for future clinical trials. Although HDAC inhibitors can function as pan-HDAC inhibitors due to their wide spectrum of action, few have been approved for lung cancer treatment. Currently, panobinostat, vorinostat, mocetinostat, valproic acid, entinostat, and chidamide, have completed phase I, II, and III clinical trials (See Table 2).

**TABLE 2:** Structure of HDACs inhibitors against deacetylase isozymes in lung cancer and clinical assays.

Structure HDAC inhibitors	*In vitro* tests NSCLC cell lines	HDAC class	HDAC isozyme	HDAC Inhibition IC50 (nM)	Clinical assays	Reference
Panobinostat (LBH-589) 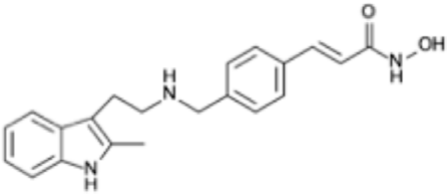	A549 Calu-1 H226 H460 H838 SKMES-1	Class I Class IIa Class IIb	HDAC-1 HDAC-2 HDAC-3 HDAC-8 HDAC-4 HDAC-7 HDAC-9 HDAC-6	4–31	Phase I Completed (NCT02890069)	Wang et al. (2018b)
Vorinostat (SAHA) 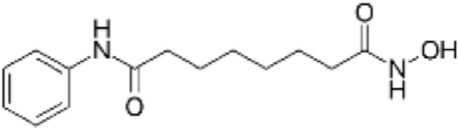	PC-14 A549 A549-Oct4 H460-luc	Class I Class IIa Class IIb	HDAC-1 HDAC-2 HDAC-3 HDAC-8 HDAC-4 HDAC-7 HDAC-9 HDAC-6	81.7 ± 1.4 281.0 ± 2.2 32.8 ± 1.6 >10 20.0 ± 0.2 ------ 1975.0 ± 35.0 ------	Phase I Completed (NCT01059552)	Shieh et al. (2017)
CG347B 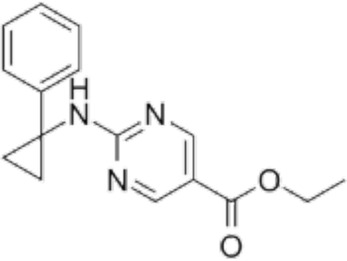	A549 NCI-H460 NCI-H1299	Class IIb	HDAC-6	5000	Not yet evaluated	Wang et al. (2020b)
Pracinostat 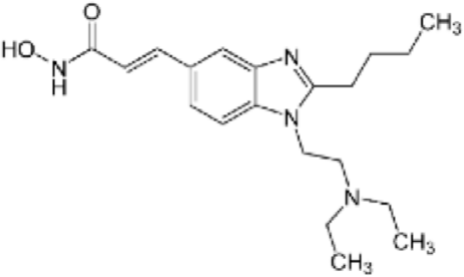	LU505, DMS53	Class I Class IIa Class IIb Class IV	HDAC-1 HDAC-2 HDAC-3 HDAC-8 HDAC-4 HDAC-7 HDAC-9 HDAC-6 HDAC-11	125	Not yet evaluated	Jia et al. (2018)
Trichostatin A (TSA) 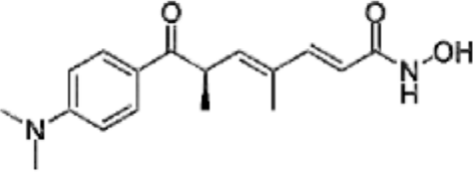	H1975 HCC827 A549	Class I Class IIb	HDAC-1 HDAC-2 HDAC-3 HDAC-6	81.3 232 400	Not yet evaluated	Tang et al. (2018), Meng et al. (2021)
CAY10603 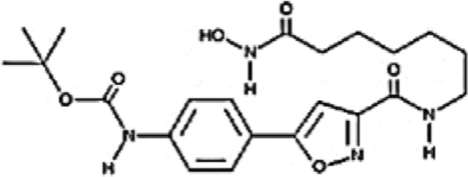	A549 HCC827 H1975 C57BL/6 male mice	Class IIb	HDAC-6	10, 5 mg/kg	Not yet evaluated	Liu et al., 2019, Wang et al. (2016c)
SNOH-3 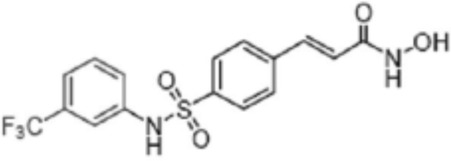	A549 NCI-H1299	Class I	HDAC-1	822	Not yet evaluated	Wang et al. (2016a)
MPT0E028 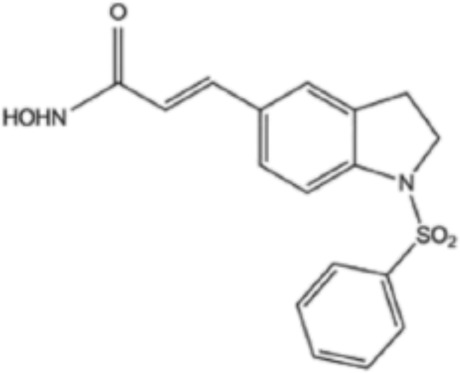	A549 H1299 H1975 PC9/IR CL97	Class I Class IIb	HDAC-1 HDAC-2 HDAC-8 HDAC-6	1550 ± 140 1100 ± 20 1300 ± 130 1660 ± 410 1350 ± 110	Not yet evaluated	Chen et al. (2013)
S11 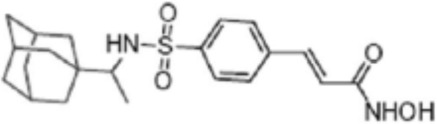	A549 CDDP resistants H460 CDDP resistants H1299 CDDP resistants	Class I	HDAC-1 HDAC-2	1380 2320 139O	Not yet evaluated	Sun et al. (2019)
Mocetinostat 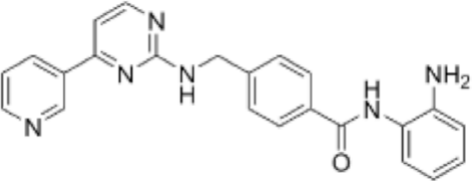	H23 H1299 H1437 H1703 H1792 H1838 H2122 CT26	Class I	HDAC-1 HDAC-2 HDAC-3 HDAC-8	1000	Phase II completed (NCT02954991)	Briere et al. (2018)
Valproic acid (VPA) 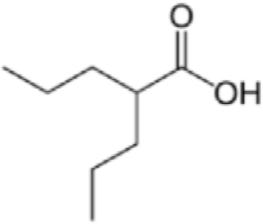	1. A549	Class I Class IIa	HDAC-1 HDAC-2 HDAC-3 HDAC-8 HDAC-4 HDAC-5 HDAC-7 HDAC-9	10.5 6.8 and 4.5	Phase I-II completed (NCT00084981, NCT00759824)	Kalantar et al., 2021
CUDC-907 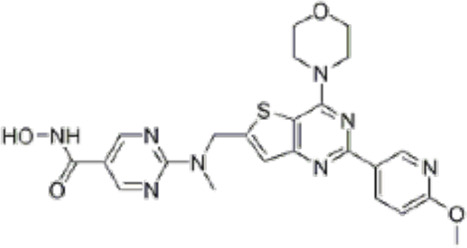	H196 SHP77 DMD273 H446 H526 H69 H82 DMS79 H2066	Class I Class IIa Class IIb	HDAC-1 HDAC-2 HDAC-3 HDAC-8 HDAC-4 HDAC-5 HDAC-7 HDAC-9 HDAC-6 HDAC-10	0.49 3.91 3.55 1.69 1.00 2.22 2.22 8.83 0.70	Not yet evaluated	Ma et al. (2020)
Selisistat (EX527) 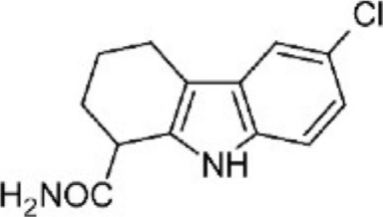	A549 H1299 H157 DMS53 and Calu-1 cell lines	Class III	SIRT	10,000 5000	Not yet evaluated	Chen et al. (2017a), Guo et al. (2021)
YF454A 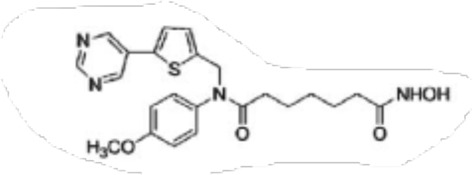	A549 H1299 H1975 and PC9			150 to 3500	Not yet evaluated	Yu et al. (2017)

Refs (Chen et al., 2013; Wang L. et al., 2016; Wang et al., 2016c; Chen G. et al., 2017; Shieh et al., 2017; Yu et al., 2017; Wang L. et al., 2018; Jia et al., 2018; Tang et al., 2018; Luo et al., 2019; Sun et al., 2019; Ma et al., 2020; Zhang et al., 2020; Guo et al., 2021; Meng et al., 2021).

Although HDAC inhibitors mainly target different classes of HDACs, their inhibitory effects are not limited to these enzymes. This is due to the deregulation of multiple targets and the great promiscuity some HDACs show. Moreover, the affinity of HDAC inhibitors relies on several factors, such as posttranslational modifications, or the composition of HDAC complexes. So the specificity of HDAC inhibitors can be limited. A recent study by Lechner et al. (2022), which was based on proteomic assays, showed that some HDAC inhibitors have off-target substrates and low selectivity, particularly HDAC-6 inhibitor tubastatin A. The authors also showed that HDAC inhibitor hydroxamic acid has as an unexpected target the metallobeta-beta-lactamase-domain-containing protein 2 (MBLAC2), which leads to the accumulation of extracellular vesicles *in vitro*. Although these off-targets deserve further research to identify unknown substrates, HDAC inhibitors are still considered promising drugs that can help improve treatments for lung cancer, as shown *in vitro*, *in vivo*, and in clinical assays.

## 6 Modulation of HDACs by natural compounds in lung cancer

**FIGURE 4:**
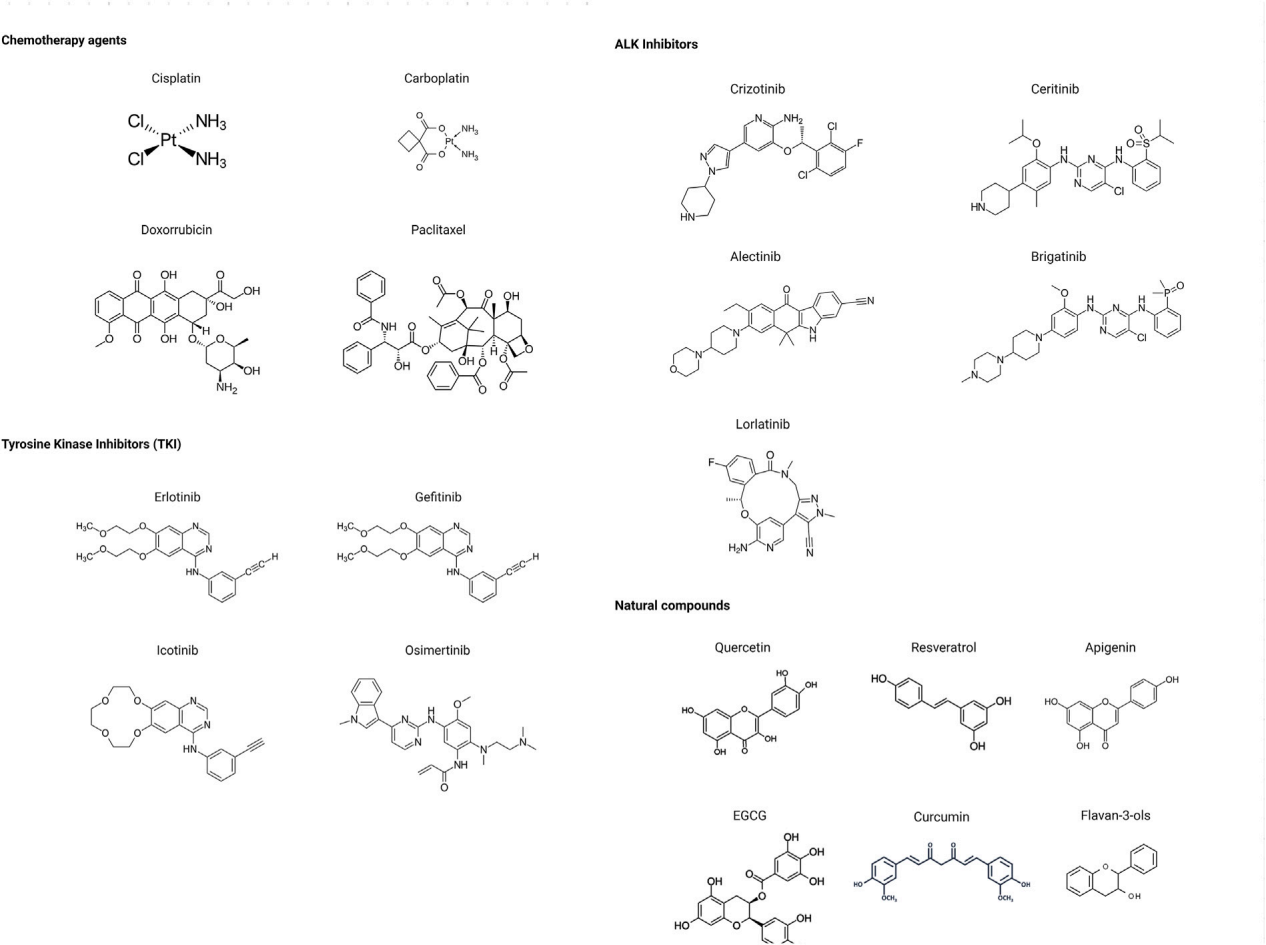
Chemical structure of chemotherapeutic agents, TKIs, ALK inhibitors, and phytochemicals discussed in the text.

Many phytochemicals play critical roles in the treatment of various diseases, including cancer. Phytochemicals are compounds derived from plants. Either purified or obtained as extracts, they are of great interest because of their therapeutic or chemopreventive properties (Lin et al., 2020). Phytochemicals include a wide range of secondary metabolites such as polyphenols, flavonoids, steroid saponins, organosulfur compounds, and vitamins. Many phytochemicals have great antioxidant potential, so they can be chemopreventive agents. Besides, their use might be beneficial in combination with established conventional treatments (Forni et al., 2019; Lin et al., 2020). Although the bioavailability of phytochemicals when ingesting foods that contain them (fruits, vegetables, and whole grains) is low, some foods have the concentrations required to exert an anticancer effect. The structure of the phytochemicals discussed in the present review and chemotherapeutic agents and TKIs are shown in Figure 4.

**FIGURE 5:**
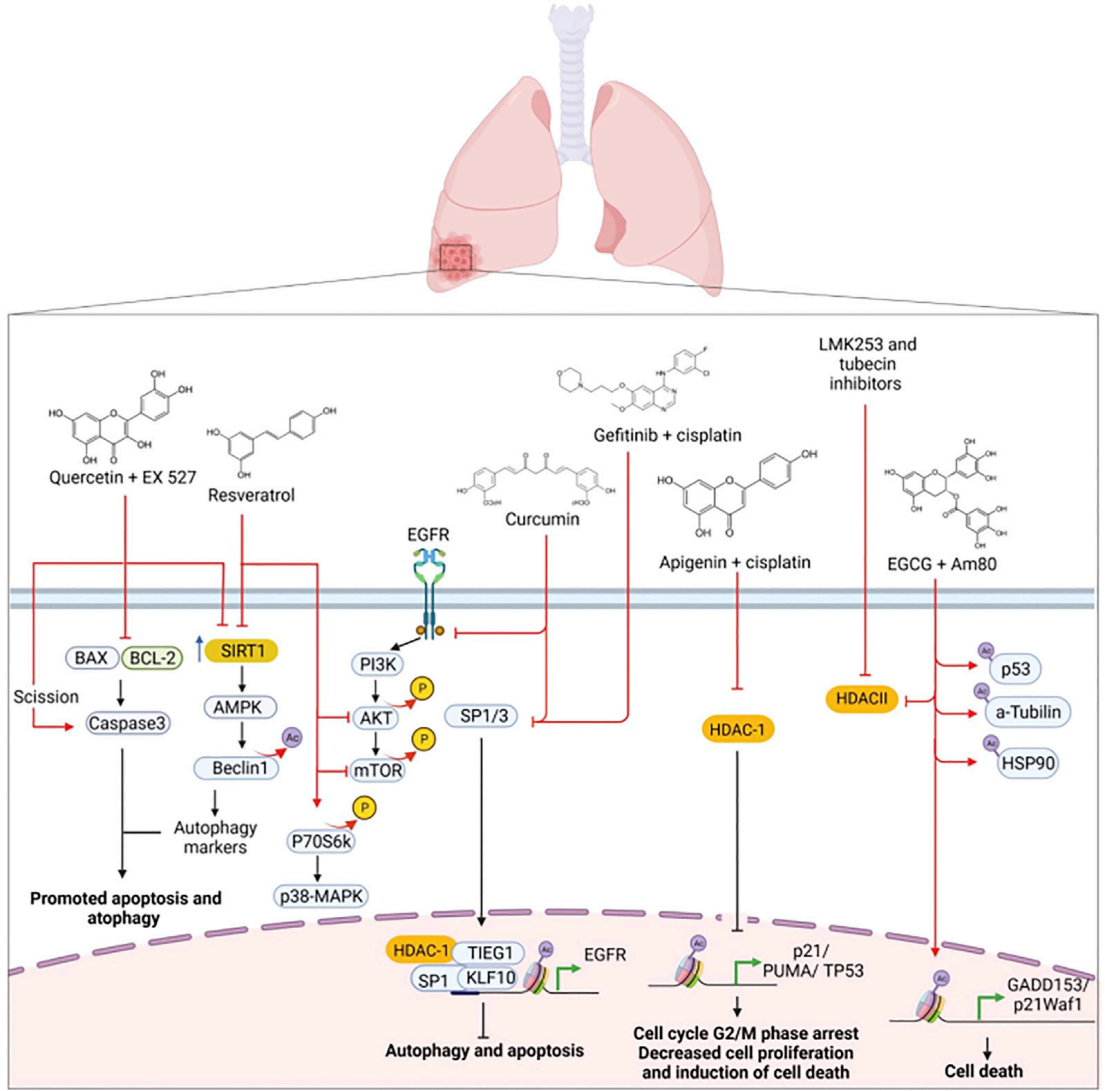
Modulation of HDACs by phytochemicals plus standard therapy in lung cancer. 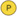
Phosphorylation 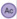
Acetylation 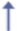
Up-regulation 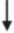
Activation 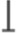
Inhibition 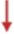
Mechanism promoted by treatments

Further studies show that some phytochemicals act as critical modulators of HDACs in lung cancer resistant to standard therapies (Figure 5). For example, curcumin is a potent anti-inflammatory, and an antioxidant compound found in high concentrations in spice turmeric, which gives curry its yellow appearance. Curcumin is a phenolic acid that also shows antitumor effects and alters the expression of Sp transcription factors. Sp transcription factors are members of the Sp/Krppel-like family (KLF) conformed by Sp1, Sp2, Sp3, and Sp4 (Hewlings and Kalman, 2017). These Sp proteins are overexpressed in several tumors, including lung cancer. Sp1 transcription factor and HDAC-1 regulate EGFR expression through the complex with Kruppel-like factor 10 (KLF10/TIEG1), which binds to promoter sites within Sp1 to inhibit histone acetylation and repress Sp1 transcription (Beishline and Azizkhan-Clifford, 2015).

Chen et al. (2019) investigated the effect of curcumin on abrogating gefitinib resistance of NSCLC cells with wild-type EGFR and KRAS mutations using *in vitro* and *in vivo* models. Gefitinib-resistant NSCLC cell lines (H157 and H1299) treated with a combination of curcumin and gefitinib diminish the activity of EGFR through repression of Sp1, which in turn blocks the interaction of Sp1 with HDAC-1. Furthermore, curcumin and gefitinib synergistically inhibit the receptor tyrosine kinase signaling and ERK-AKT pathways. Whereas they induce autophagic cell death, and autophagy-mediated apoptosis, by suppressing the interaction of Sp1/EGFR. Using xenograft mouse models, the authors showed that the combination of gefitinib and curcumin significantly inhibits tumor growth compared to treatment with either drug alone.

Apigenin is a flavone found in vegetables such as fresh parsley, vine spinach, celery seed, green celery heart, Chinese celery, and dried oregano. This flavone has multiple properties, being a powerful antioxidant and possessing antimutagenic, anticarcinogenic, and anti-inflammatory activities (Wang et al., 2019). Yan et al. showed that apigenin promotes arrest in the G2/M cell cycle in lung cancer cell lines by reducing the binding of HDAC-1. This results in histone H3 acetylation of the promoters for p21 and PUMA genes which increases the association of RNA polymerase II and Sp1 (Yan et al., 2020). Apigenin can also induce acetylation of the TP53 promoter. The combination of cisplatin with apigenin showed a remarkable prolongation of S-phase and arrest in G2/M-phase, a decrease in cell proliferation, and induction of cell death. Thus, apigenin enhances the anticancer activity of cisplatin (Wang et al., 2019). However, apigenin is insoluble in polar solvents such as water, which further hampers pharmacokinetic studies. Its absorption, distribution, metabolism, and excretion are slow during phase II metabolism in the gastrointestinal tract. Apigenin is poorly absorbed because of its insolubility, moderate permeability, chemical instability, and the conjugation reactions of glucuronidation and sulfonation (Meyer et al., 2006; Telange et al., 2017; Wang et al., 2019; Yan et al., 2020). These disadvantages can be a barrier to an effective therapeutic strategy.

Flavan-3-ols (flavanols or catechins) are phytochemicals that also modulate HDACs and are found in green tea. Epigallocatechin gallate (EGCG), epicatechin, epicatechin gallate, and catechin are found in green tea extracts, but they are also found in cocoa, prune juice, broad bean pods, acaí oil, and argan oil. EGCG has chemopreventive, anticarcinogenic, antiapoptotic, and anti-inflammatory properties in cancer (Xu et al., 2021). Oya et al. showed that the combination of EGCG and Am80, a synthetic retinoid, synergistically induced apoptosis of the lung cancer cell line PC-9. EGCG and Am80 increase the expression of growth arrest and DNA damage-inducible gene 153 (GADD153), death receptor 5, and p21waf1 genes. The synergistic mechanistic effect includes the increased acetylation of non-histone proteins p53, α-tubulin, and HSP90. This is mediated by reduced activity of class II HDACs (HDAC-4, -5, and -6) in the cytosol. Furthermore, suppression of HDAC-4 and -5 increases p21waf1 expression, while suppression of HDAC-6 promotes high expression of GADD153 and p21waf1, leading to cell death. Overall, EGCG, combined with Am80 changes acetylation levels of non-histone proteins *via* downregulation of HDAC4, -5, and -6 and stimulates apoptotic induction in lung cancer cell line PC-9 (Oya et al., 2017).

Resveratrol (trans-3,4,5-trihydroxystilbene) is a natural polyphenol found in large amounts in the root of Japanese knotweed (Polygonum cuspidatum). Resveratrol is also found in peanuts, red grapes, pistachios, red and white wine, blueberries, cranberries, peas, soybeans, and even cocoa and dark chocolate. This phytochemical has anticarcinogenic, neuroprotective, cardioprotective, and nephroprotective properties (Shrikanta et al., 2015; Vervandier-Fasseur and Latruffe, 2019). While quercetin is a flavonol with a wide range of properties such as antioxidant, anti-inflammatory, antibacterial, antiviral, gastroprotective, and immunomodulating activities. Quercetin is found in apples, citrus fruits, berries, dark cherries, cranberries, onions, broccoli, cabbage, raw asparagus, peppers, legumes, whole grains, and capers, among others (Ferraz et al., 2021). Wang et al. (2018a) found that resveratrol has antitumor effects by inhibiting cell proliferation and promoting cell apoptosis in NSCLC cell lines A549 and H1299. Mechanistically, resveratrol treatment increases SIRT1, Beclin1, and LC3 II/I, while decreasing p62 expression. Overexpression of SIRT1, which promotes Beclin1 deacetylation, leads to autophagosome formation, suggesting that resveratrol might induce autophagy. Furthermore, treatment with resveratrol inhibits Akt/mTOR and activates p38 MAPK in NSCLC cells. Thus, resveratrol inhibits proliferation and induces apoptosis and autophagy *via* inhibition of Akt/mTOR and activation of the p38 MAPK signaling pathway. Resveratrol-induced autophagy may act as a protective mechanism to promote NSCLC cell survival (Wang et al., 2018a). Similarly, Guo et al. (2021) reported that quercetin induces proapoptotic autophagy via the SIRT1/AMPK signaling pathway in A549 and H1299 lung cancer cell lines. They found that quercetin increased the levels of SIRT1 protein and the pAMPK-AMPK ratio, as well as LC3-II, Beclin 1, Atg5, Atg7, and Atg12 mRNA levels.

Some drugs used as therapies against cancer, originate from or are based on phytochemicals. For example, romidepsin (FK228) is a selective inhibitor of HDACs that received approval from the US Food and Drug Administration (FDA) in 2009. Romidepsin is typically used to treat cutaneous T-cell lymphoma. This compound was tested in phase II clinical trials in patients with NSCLC. However, romidepsin had low clinical efficacy in advanced stages of NSCLC and most patients showed disease progression. Similar results were found in patients with limited and extensive stages of SCLC treated with romidepsin. Another phase I clinical trial treated NSCLC patients with romidepsin plus erlotinib. The results did not show benefits for the study population (Schrump et al., 2007, 2008; Amiri-Kordestani et al., 2013). In summary, despite romidepsin being a specific inhibitor of HDAC in other types of cancer, no beneficial effects are observed for lung cancer patients.

## 7 Conclusion and perspectives

Despite significant advances in lung cancer therapies, acquiring mutations in critical genes such as EGFR, Kras, and c-Met, among others, favors resistance to standard therapies such as cisplatin and TKIs. The heterogeneity and hostility within the tumor microenvironment favor the selection of certain subpopulations of tumor cells, such as cancer stem cells. These cells express more aggressive, invasive, and multidrug-resistant phenotypes. Therefore, new and accessible options for the treatment of lung cancer are needed.

HDACs are promising targets as they are involved in tumorigenesis, tumor progression, metastasis, and resistance to lung cancer therapies. Therefore, the development of specific inhibitors against the different HDAC classes is encouraging as these inhibitors have demonstrated efficacy *in vitro* and *in vivo* against lung cancer.

About 80% of the drugs approved by the FDA used as therapies against cancer originate from or are based on phytochemicals. These compounds exert synergistic effects to increase the effectiveness of chemotherapeutic drugs. Some of these phytochemicals regulate the activity of HDACs. Recent studies have demonstrated that these compounds possess antitumorigenic properties in lung cancer. These properties include 1) repair of epigenetic changes, 2) induction of cell death, 3) chemopreventive effects, and 4) restoring the susceptibility of resistant tumor cells to chemotherapeutic drugs.

There is still scarce evidence of the pharmacokinetics and pharmacodynamics of phytochemicals in lung cancer. Preliminary studies of some phytochemicals have shown that these compounds have poor bioavailability, and their pharmacokinetic profiles have limited their use in cancer therapy. However, the combination of phytochemicals that act as HDAC inhibitors with standard therapies has synergistic effects against lung cancer (Figure 5). Thus, understanding how phytochemicals modulate the resistance to therapy through HDACs and the effects produced in combination with standard therapies is a potential area that deserves further research.
